# Indexed Left Atrial Adipose Tissue Area Is Associated With Severity of Atrial Fibrillation and Atrial Fibrillation Recurrence Among Patients Undergoing Catheter Ablation

**DOI:** 10.3389/fcvm.2018.00076

**Published:** 2018-06-19

**Authors:** Saket R. Sanghai, Mayank Sardana, Barinder Hansra, Darleen M. Lessard, Seth T. Dahlberg, Gerard P. Aurigemma, Timothy P. Fitzgibbons, David D. McManus

**Affiliations:** ^1^Division of Cardiovascular Medicine, Department of Medicine, University of Massachusetts Medical School, Worcester, MA, United States; ^2^Department of Biostatistics and Health Service Research, University of Massachusetts Medical School, Worcester, MA, United States

**Keywords:** atrial fibrillation, epicardial adipose tissue, cardiac CT, catheter ablation, recurrence, left atrial remodeling

## Abstract

**Background:** Epicardial adipose tissue (EAT) has been associated with adverse left atrial (LA) remodeling and atrial fibrillation (AF) outcomes, possibly because of paracrine signaling.

**Objectives:** We examined factors associated with a novel measure of EAT i.e., indexed LAEAT (iLAEAT) and its prognostic significance after catheter ablation (CA) of atrial fibrillation (AF).

**Methods:** We performed a retrospective analysis of 274 participants with AF referred for CA. LAEAT area was measured from a single pre-ablation CT image and indexed to body surface area (BSA) to calculate iLAEAT. Clinical, echocardiographic data and 1-year AF recurrence rates after CA were compared across tertiles of iLAEAT. We performed logistic regression analysis adjusting for factors associated with AF to examine relations between iLAEAT and AF recurrence.

**Results:** Mean age of participants was 61 ± 10 years, 136 (49%) were women, mean BMI was 32 ± 9 kg/m^2^ and 85 (31%) had persistent AF. Mean iLAEAT was 0.82 ± 0.53 cm^2^/m^2^. Over 12-months, 109 (40%) had AF recurrence. Participants in the highest iLAEAT tertile were older, had higher CHA_2_DS_2_VASC scores, more likely to be male, have greater LA volume, and were more likely to have persistent (vs. paroxysmal) type AF than participants in the lowest iLAEAT tertile (*p* for all < 0.05). In regression analyses, iLAEAT was associated with higher odds of AF recurrence (OR = 2.93; 95% CI 1.34–6.43).

**Conclusions:** iLAEAT can quantify LA adipose tissue burden using standard CT images. It is strongly associated with AF risk factors and outcomes, supporting the hypothesis that EAT plays a role in the pathophysiology of AF.

## Introduction

Population-based studies have demonstrated that visceral adipose tissue is strongly associated with cardiovascular disease including coronary atherosclerosis, carotid stenosis, heart failure and atrial fibrillation (AF) (1–6). This is partly due to the close relationship between visceral adiposity and increased burden of adverse metabolic factors, such as diabetes mellitus and hypertension (7, 8). Imaging techniques such as echocardiography, cardiac computed tomography (CCT) and magnetic resonance imaging provide a more precise assessment of visceral adiposity (9–11) than anthropometric measures such as body mass index (BMI) and waist circumference (12). Epicardial adipose (EAT) tissue is the visceral adipose depot of the heart. It is directly adjacent to the myocardium, contained within the visceral pericardium (13). Periatrial adipose tissue is tightly linked to markers of atrial remodeling and vulnerability to AF, perhaps due to paracrine and/or vasocrine effects (14–18) and/or interactions with the cardiac inputs of the autonomic nervous system (19, 20). Recent studies have shown that atrial adipose tissue relates to recurrence of AF after catheter ablation (11, 21–24). However, it remains unknown whether it confers risk independent of total body mass or visceral adiposity. To overcome these limitations, we propose to calculate an indexed left atrial EAT (iLAEAT) derived by adjusting LA EAT measured on quantitative CCT based on body surface area (BSA). Using data from a prospective treatment registry including AF patients undergoing catheter ablation, CCT and echocardiographic cardiac phenotyping, we sought to examine associations between an iLAEAT, parameters of adverse cardiac remodeling, as well as type of AF and recurrence of AF after ablation.

## Methods

### Patient population

We reviewed the medical records of 420 consecutive participants with AF who were referred to the University of Massachusetts Medical Center (UMMC) Atrial Fibrillation Clinic for AF ablation between November 2011 to April 2016. All participants underwent a pre-ablation CCT scan and an echocardiogram within 3 months of the procedure. Late AF recurrence was adjudicated for 312 patients. Twenty-nine were excluded due to significant mitral valve pathology on echocardiography to restrict the sample to subjects with true “non-valvular” AF. Nine subjects were excluded since their CCT scans were not available for review. Thus, 274 were included in the final analysis. Participants were prospectively studied to determine recurrence of AF after ablation. The study was approved by the University of Massachusetts Medical School Institutional Review Board (IRB#00003865).

### Clinical variables

Demographic information, including age, gender and race were abstracted from the health record by trained study staff. Clinical covariates abstracted included diabetes mellitus, hypertension, dyslipidemia, heart failure, coronary artery disease, obstructive sleep apnea, stroke and peripheral vascular disease. Trained staff calculated the CHA_2_DS_2_VASC score for each patient using the following variables- congestive heart failure (or Left ventricular systolic dysfunction), hypertension, age 65–74 years (1 point) age ≥ 75 years (2 points), diabetes mellitus, prior stroke or TIA or thromboembolism (2 points), vascular disease (e.g., peripheral artery disease, myocardial infarction, aortic plaque) and gender (i.e., female sex) (25). The burden of pre-existing AF was classified at the time of the index ablation by trained electrophysiology fellows or advanced care practitioners who assisted with the procedure [3 categories: paroxysmal (at least 1 day of AF but < 7 consecutive days of AF), persistent (at least 7 consecutive days with >23 h of AF) or long-standing persistent (>6 months of AF)]. BSA was calculated for each patient using their documented height and weight at the time of the CCT imaging using the Mosteller formula (26).

### Catheter ablation for atrial fibrillation

All patients enrolled in the UMMC AF registry underwent catheter ablation using radiofrequency ablation or cryoablation by one out of five board certified electrophysiologists. A standardized approach was used in almost all cases. In patients who had a radiofrequency ablation procedure, a wide area circumferential ablation was performed with an open irrigated or 8 mm radiofrequency ablation catheter. For cryoablation procedures, pulmonary vein isolation was performed using the Arctic Front cryoballoon catheter (28 or 23 mm, Medtronic Inc. Minneapolis, MN). Cryoablation was done twice in each pulmonary vein for 150–240 s. Both entry and exit block was confirmed using a circular mapping catheter. Patients with persistent AF received additional lesions at the left atrial roof, the basal posterior wall, and the left atrial isthmus at the discretion of the operating electrophysiologist. Anti-arrhythmic medications were stopped at the discretion of the treating electrophysiologist if there was no symptomatic or electrocardiographic evidence of AF recurrence. Oral anticoagulation with warfarin or direct oral anticoagulant was continued for at least 1 month as determined by the treating physician, typically based on the participant's baseline CHA_2_DS_2_VASC risk score.

### Echocardiographic measurements

Left ventricular (LV) and LA volumes were measured according to the American Society of Echocardiography guidelines for chamber quantification (27). Pulsed wave tissue Doppler imaging was used to acquire mitral inflow velocity (E) and mitral annular velocities (e') to estimate LV filling pressures (E/e') (28). Right ventricular (RV) systolic function was assessed by calculating fractional area change in the RV- focused apical 4-chamber view. All measurements were averaged over 3 beats when AF was present (29). Cardiac chamber dimensions as well as LV ejection fraction were acquired directly from the echocardiogram reports. Doppler velocities and RV fractional area change were measured by one of the authors (SRS). The intra-observer reproducibility was *r* = 0.94.

### EAT quantification

**Figure 1:**
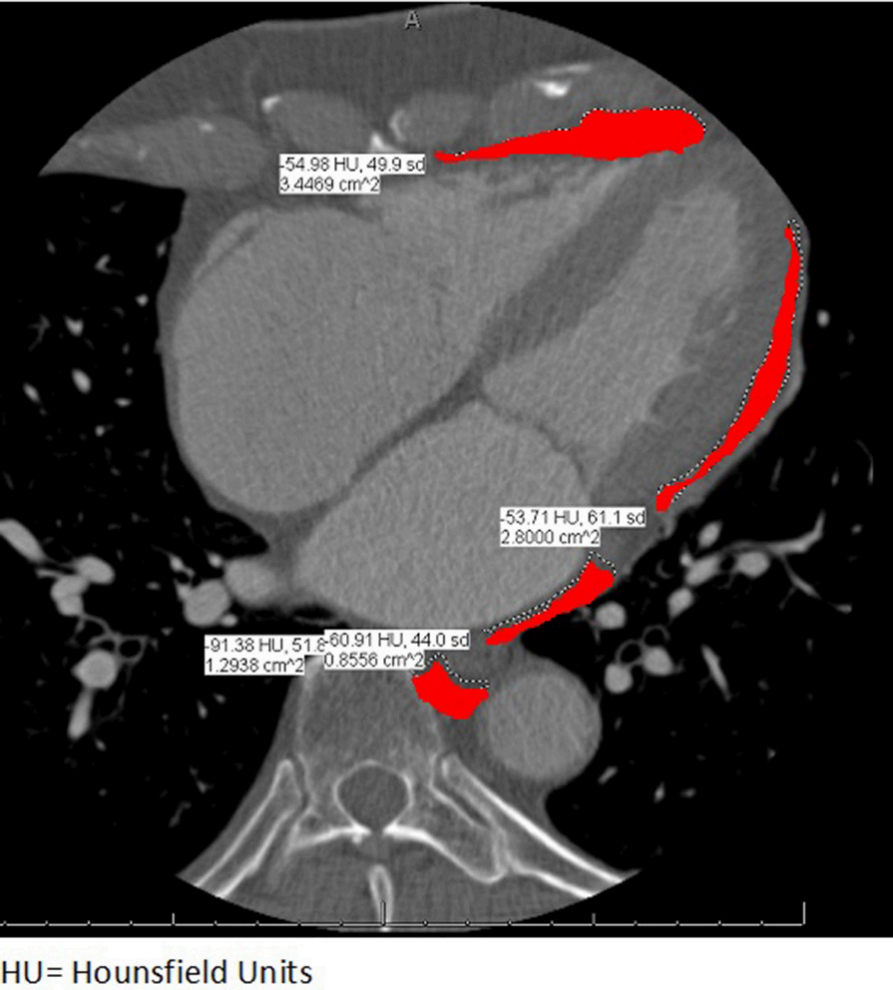
Example of a study participant showing EAT measurement in a single CT image using a 4-chamber equivalent view. Areas in red depict traced EAT with their respective areas and Hounsfield Units.

CCT imaging was performed using a Siemens Somatom Definition Flash 128 slice dual source CT Scanner. Unenhanced scans were acquired with a high-pitch FLASH protocol. Contrast enhanced scans were acquired using a sequential acquisition protocol with injection of low ionic contrast. Contrast timing was calculated using a 15 ml contrast test bolus followed by 50 ml saline at 5–6 ml/s. EAT area was measured by CCT in a single cross section using a 4-chamber equivalent view placing the true LV apex in view. Areas of EAT surrounding the LA, RA, LV, and RV were separately assessed by tracing regions of interest (ROI) containing heart and EAT (Figure 1). ROI's were placed at the visceral epicardium to exclude pericardial fluid. This was done by two authors (SRS and BH). Separate values for EAT surrounding the four chambers were recorded and added to obtain total EAT. The upper threshold limit of CCT attenuation for the identification of EAT was defined at (-) 43 Hounsfield units (HU) for unenhanced scans and at (-) 15 HU for contrast enhanced scans as described previously (10).

### Outcomes adjudication

All participants were followed for at least 12 months following their ablation procedure according to a standard protocol. Recurrence of AF was determined from all available clinic and hospital notes, 12 lead electrocardiograms, and cardiac monitor data. Twelve-lead electrocardiograms were ordered at 1, 3, 6, and 12 months and a 7-day cardiac event monitor was typically prescribed at 3-months after the procedure. Additional ECGs and monitor recordings were obtained if the participant had any symptoms suspicious of AF (such as chest pain, palpitations, shortness of breath). Recurrence of AF was defined as any evidence of AF on a 12 lead ECG or greater than 20 s of AF on a cardiac monitor and was confirmed by an electrophysiologist. Any electrical or pharmacologic cardioversion or repeat ablation within the follow up period was characterized as AF recurrence. Any recurrence of AF after the initial blanking period of 3 months, were considered as significant (30).

### Statistical analysis

Participants were divided into tertiles based on their iLAEAT measures reported as low, intermediate and high. This was done to identify participants at the highest risk for AF recurrence post ablation thus providing a clinically relevant risk predictor. We examined the relationship between iLAEAT tertiles and demographics, medical history, echocardiographic and biochemical factors. We used analysis of variance and chi-squared statistic to test for difference in continuous and categorical factors respectively among the tertiles. Multivariable logistic regression analysis was used to examine the relationship of clinical, electrocardiographic and echocardiographic predictors with type of AF and AF recurrence. Co-variates used in the multivariable models were selected *a priori* based on previously known associations with AF severity, AF type and AF recurrence (BMI, CHA_2_DS_2_VASC, E/e', LA volume, LV mass index). CHA_2_DS_2_VASC score was used in place of individual component variables to avoid overfitting of the regression model. Subsequently, we conducted several stratified analyses to examine iLAEAT relations with AF recurrence after CA among participants of different ages, genders, types of AF and based on their BMI. We considered *p* < 0.05 as significant. Statistical analyses were performed using MedCalc (v16.2.1.0-64 bit, MedCalc software, Mariakerke, Belgium) and SAS (9.4) software.

## Results

The baseline characteristics of all registry participants who had AF ablation during the study period and current study participants are shown in Table 1. The mean age of the study cohort was 61 ± 10 years, 136 (49%) were female, mean BMI was 32 ± 9 kg/m^2^ and cohort participants had a mean CHA_2_DS_2_VASC of 2.2 ± 1.5, suggesting that majority of the participants had moderate or greater burden of cardiovascular comorbidities. Eighty-five participants (31%) had persistent AF and 239 (87%) were on anti-arrhythmic drugs before ablation. The mean LV ejection fraction among participants was 58 ± 7% and the mean LA volume was 87 ± 29 ml. Most participants 195 (72%) underwent cryoablation and the remainder underwent radiofrequency ablation. Over a 12-month period of standardized, intensive follow up, 109 (40%) of participants had at least one recurrence of AF. Of these, 48 individuals (44%, *n* = 109) underwent a repeat ablation and 28 underwent (26%, *n* = 109) a cardioversion.

**Table 1:** Baseline characteristics of All Registry Participants and Study participants.

**Variables**	**All registry participants (*n* = 420)**	**Study participants (*n* = 274)**
Age, years	61 ± 10	61 ± 10
Female sex	184 (44)	136 (49)
Systolic BP, mmHg	128± 18	127 ± 16
BMI, kg/m^2^	32 ± 16	32 ± 9
Current smoker	54 (13)	38 (14)
Persistent AF	123 (29)	85 (31)
CHA_2_DS_2_VASC score	2.1 ± 1.4	2.2 ± 1.5
Prior antiarrhythmic drugs	373 (89)	239 (87)
Hypertension	300 (71)	195 (71)
Diabetes	81 (19)	56 (20)
Heart Failure	68 (16)	33 (12)
CAD	85 (20)	62 (22)
Obstructive sleep apnea	156 (37)	108 (39)
Stroke/TIA	20 (5)	18 (6)
PAD	47 (11)	30 (11)
Cryo ablation	295 (70)	195 (72)
Late AF recurrence^†^	123 (39)	109 (40)

*Continuous variables are presented as mean ± standard deviation and categorical variables are presented as n (%). AF, Atrial Fibrillation; BMI, Body Mass Index; BP, Blood Pressure; CAD, Coronary Artery Disease; PAD, Peripheral Arterial Disease; TIA, Transient Ischemic Attack*.

† *No. of patient in whom AF recurrence outcome was adjudicated was 312*.

The inter and intra-observer reproducibility for EAT measurement was (*r* = 0.89 and 0.95), respectively. For 10 randomly selected study participants, we measured the time required for measuring EAT surrounding the LA, LV, and RV separately. The median time required for reporting EAT for an experienced operator (>100 prior measurements) was 71 s (range 55–92 s).

Mean iLAEAT among participants was 0.82 ± 0.53 cm^2^/m^2^. Participants in the high tertile of iLAEAT when compared to participants in the low iLAEAT tertile tended to be older (63 ± 9 vs. 60 ± 10, *p* = 0.04), have a higher mean CHA_2_DS_2_VASC score (2.6 ± 1.5 vs. 2.0 ± 1.4, *p* < 0.01), were less likely to be female (46% vs. 61%, *p* = 0.04), and more likely to have persistent AF (43.9% vs. 14.3%, *p* < 0.01) (Table 2A).

**Table 2A:** Baseline clinical characteristics of study participants stratified by iLAEAT.

**Variables**	**Lowest iLAEAT tertile (*n* = 92) (0–0.51)**	**Intermediate iLAEAT tertile (*n* = 91) (0.52–0.94)**	**Highest iLAEAT tertile (*n* = 91) (0.95–2.83)**	***P*-value**
Mean iLAEAT	0.31 ± 0.14	0.72 ± 0.13	1.42 ± 0.42	–
Age, years	60 ± 10	60 ± 11	63 ± 9	0.04
Female sex	56 (61)	40 (44)	42 (46)	0.04
Systolic BP, mmHg	127 ± 20	127 ± 16	132 ± 19	0.09
BMI, kg/m^2^	32 ± 12	32 ± 6	32 ± 6	0.96
Current smoker	35 (38)	32 (35)	40 (70)	0.3
Persistent AF	13 (14)	32 (35)	40 (44)	< 0.01
CHA_2_DS_2_VASC score	2 ± 1.4	2 ± 1.5	2.6 ± 1.5	< 0.01
Prior antiarrhythmic drugs	80 (87)	80 (88)	79 (87)	0.97
Hypertension	56 (36)	64 (70)	75 (82)	< 0.01
Diabetes	18 (20)	12 (13)	26 (29)	0.03
Heart Failure	6 (7)	10 (11)	17 (19)	0.04
CAD	18 (20)	15 (17)	29 (32)	0.03
Obstructive sleep apnea	34 (37)	37 (41)	37 (41)	0.84
Stroke/TIA	6 (7)	5 (5)	7 (8)	0.84
PAD	12 (13)	10 (11)	8 (9)	0.64
Late AF recurrence	24 (26)	36 (40)	49 (54)	< 0.01

*Continuous variables are presented as mean ± standard deviation and categorical variables are presented as n (%). AF, Atrial Fibrillation; BMI, Body Mass Index; BP, Blood Pressure; CV, Cardioversion; CAD, Coronary Artery Disease; PAD, Peripheral Arterial Disease; TIA, Transient Ischemic Attack*.

### iLAEAT relates to echocardiographic and electrocardiographic measures of cardiac structure and function

Participants in the highest tertile of iLAEAT had a greater LA volume (90 ± 28 ml vs. 81 ± 29 ml, *p* = 0.04). The groups did not differ significantly with respect to LVEF, LV mass, diastolic function or RV function (Table 2). Similarly, sinus node recovery time and QRS duration did not differ among the three groups. The PR interval was longer in participants with highest iLAEAT (184 ± 30 ms in the highest tertile vs. 167 ± 40 ms in the lowest iLAEAT tertile, *p* = 0.02).

### iLAEAT relates to validated biomarkers of AF recurrence

A significantly greater proportion of participants in the highest iLAEAT tertile had persistent AF as compared to the lowest tertile (43.9% persistent AF in the highest iLAEAT tertile vs. 14.3% in the lowest tertile, *p* < 0.01). Higher iLAEAT was also associated with higher circulating C-reaction protein (CRP) levels (4.1 ± 1.7 mg/L vs. 2.8 ± 2.6 mg/L, *p* = 0.01) (Table 2B).

**Table 2B:** Baseline echocardiographic, electrocardiographic and biochemical differences of study participants stratified by iLAEAT.

**Variables**	**Lowest iLAEAT tertile (*n* = 92)**	**Intermediate iLAEAT tertile (*n* = 91)**	**Highest iLAEAT tertile (*n* = 91)**	***P*-value**
LVEF, %	59 ± 8	58 ± 9	58 ± 7	0.68
LVMI, gm/m^2^	83 ± 22	83 ± 25	82 ± 22	0.99
LAV, ml	81 ± 29	91 ± 28	90 ± 28	0.04
E/e'	9 ± 3	9 ± 4	10 ± 4	0.41
E/A	1.4 ± 0.6	1.8 ± 2.6	1.4 ± 1	0.35
RV FAC, %	41 ± 9	41 ± 9	42 ± 7	0.84
SNRT, ms^†^	1048 ± 436	1115 ± 806	1203 ± 641	0.59
PR duration, ms	167 ± 40	174 ± 37	184 ± 30	0.02
QRS duration, ms	103 ± 22	107 ± 65	105 ± 50	0.91
CRP mg/L ^†^	2.8 ± 2.6	6 ± 2.8	4.1 ± 1.7	0.01

† *Number of participants for which these data were available: SNRT = 53; CRP = 85. CRP, C-reactive Protein; E/A, Mitral inflow E velocity/Mitral inflow A velocity ratio; E/e' avg, Mitral inflow E velocity/average tissue Doppler e' velocity ratio; LAV, Left Atrial volume; LVEF, Left Ventricular Ejection Fraction; LVMI, Left Ventricular Mass Index; RV FAC, Right Ventricular Fractional Area Change; SNRT, Sinus Node Refractory Time*.

### iLAEAT relates to pre-ablation AF type and clinically significant AF recurrence after catheter ablation

**Figure 2:**
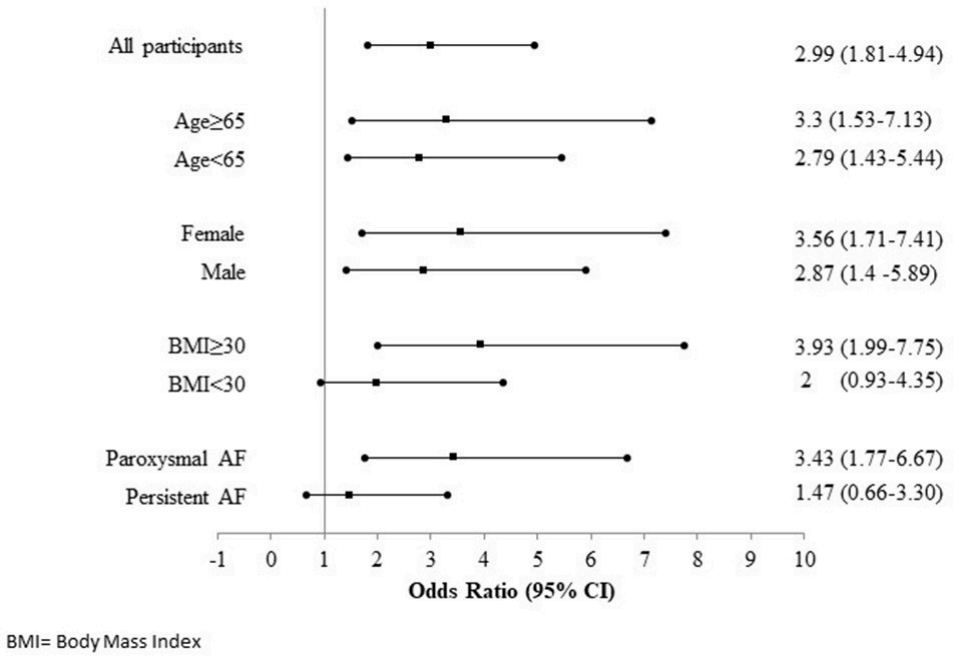
Forest plot showing relation between iLAEAT and AF recurrence stratified according to Age, Gender, BMI and AF type.

Participants in the highest iLAEAT tertile were significantly more likely to experience late AF recurrence post ablation than participants in the lowest tertile [49(53.8%) vs. 11 (12%), *p* < 0.01]. In multivariable adjusted analyses (Tables 3A,B), high iLAEAT was associated with both AF type (OR 4.5, 95% CI 1.76–11.49), and AF recurrence (OR 2.93, 95% CI 1.34–6.43). There was a trend toward greater AF recurrence among participants in the intermediate tertile of iLAEAT but did not reach statistical significance (OR 1.66, 95% CI 0.76–3.59). In stratified analyses (Figure 2), iLAEAT was associated with AF recurrence among individuals with paroxysmal AF (OR 3.43, 95% CI 1.77–6.77) as opposed to persistent AF (OR 1.47, 95% CI 0.66–3.3). However, the absolute number of patients with persistent AF in our sample was relatively low (*n* = 85). In addition, the association between iLAEAT and AF recurrence was statistically significant among participants with BMI >30 kg/m^2^, whereas iLAEAT was not significantly associated with AF recurrence among individuals with a BMI less than 30 kg/m^2^ (OR 3.93, 95% CI 1.99–7.75 vs. OR 2, 95% CI 0.93–4.35).

**Table 3A:** Association of iLAEAT with persistent AF (vs. paroxysmal AF) in multivariable regression models.

**Predictor variable**	**Unadjusted OR (95% CI)**	***P*-value**	**Model 1 OR (95% CI)**	***P*-value**	**Model 2 OR (95% CI)**	***P-*value**
Highest iLAEAT tertile	4.77 (2.33–9.77)	< 0.01	4.71 (2.28–9.74)	< 0.01	4.5 (1.76–11.49)	< 0.01
Intermediate iLAEAT tertile	3.29 (1.59–6.82)	< 0.01	3.3 (1.59–6.83)	< 0.01	2.63 (1.03–6.68)	0.04
Lowest iLAEAT tertile	1	–	1	–	1	–

*Model 1, Multivariate model adjusting for CHA_2_DS_2_Vasc score and Body Mass Index*.

*Model 2, Model 1+ LA Volume, LV mass index, average E/e'*.

**Table 3B:** Association of iLAEAT with late AF recurrence in multivariable regression models.

**Predictor variable**	**Unadjusted OR (95% CI)**	***P-*value**	**Model 1 OR (95% CI)**	***P*-value**	**Model 2 OR (95% CI)**	***P*-value**
Highest iLAEAT tertile	3.31 (1.78–6.16)	< 0.01	3.2 (1.71–6)	< 0.01	2.93 (1.34–6.43)	0.01
Intermediate iLAEAT tertile	1.86 (0.99–3.47)	0.05	1.86 (0.99–3.49)	0.05	1.66 (0.76–3.59)	0.2
Lowest iLAEAT tertile	1	–	1	–	1	–

*Model 1, Multivariate model adjusting for CHA_2_DS_2_Vasc score and Body Mass Index*.

*Model 2, Model 1+ LA Volume, LV mass index, average E/e'*.

## Discussion

In our moderately sized cohort of prospectively enrolled subjects undergoing index ablation for symptomatic AF, we observed that a novel CCT-based measure of epicardial adipose tissue, iLAEAT, was significantly associated with echocardiographic measures of pathological LA remodeling, burden of pre-existing AF, and greater likelihood of clinically significant AF recurrence over a 1-year follow-up period. Our study further supports the hypothesis that epicardial adipose tissue plays an important role in the pathophysiology and natural history of AF and may serve as a phenotype of great use in predicting arrhythmia-free survival after catheter ablation.

Epicardial fat is a potential contributor to atrial inflammation and pathological remodeling.

EAT is a metabolically active organ that generates various bioactive substances (e.g., leptin, nitric oxide) involved in endothelial vasodilation, inhibition of vascular smooth muscle proliferation, and inhibition of acetylcholine-mediated vasoconstriction (13). Excess EAT promotes inflammation via paracrine effects (i.e., through tumor necrosis factor-alpha, interleukin-6, and other cytokines) (8). Using data from the Framingham Heart Study, we found that circulating adipocytokines relate to pathological cardiac remodeling on echocardiography (14). Specifically, higher circulating levels of adiponectin and resistin, cytokines secreted by visceral fat, are associated with lower LV mass, larger LA size, and lower LV fractional shortening. The fact that CRP levels were greater in participants in the highest tertile of iLAEAT, suggests that epicardial fat is associated with local and, possibly, systemic inflammation. Although epicardial fat may have direct local inflammatory effects, we hypothesize that patients in the highest tertile of iLAEAT had greater visceral adiposity, which is turn is more likely to be responsible for systemic inflammation (i.e., elevated CRP) in this group. Epicardial fat thickness correlates very closely with visceral adiposity and has been proposed as a simple non-invasive imaging biomarker of cardiometabolic risk for this reason (8).

### Fat adjacent to the LA also influences LA electromechanical function

In a group of 1946 Framingham Heart Study participants, total pericardial fat on CCT was associated with atrial conduction defined by P wave indices (31). Other studies showed good correlation between total pericardial fat volume and regions of complex fractionated atrial electrograms (32) as well as epicardial fat volume correlated well with high dominant frequency regions (33) thus contributing to the maintenance of AF. The strong relations between iLAEAT and pathological atrial electrophysiological remodeling explain why participants in the highest tertile of iLAEAT in our study had longer PR intervals than those in the lowest tertile.

### iLAEAT, AF burden and recurrence

Prior studies have shown that epicardial fat relates to recurrence of AF after catheter ablation (11, 21–24). However, it remains unknown whether it confers risk independent of total visceral adiposity since prior studies did not adjust for body mass. In one such study, 110 participants underwent periatrial and periventricular fat measurement on cardiac MRI using proprietary software. They showed that both measures were strongly associated with severity of AF and short-term recurrence after ablation (11). Apart from the difference in imaging, these investigators used a 3-month cut off for outcomes adjudication as compared to the 1-year follow-up period employed in our study. In another study of 600 participants undergoing radiofrequency ablation and an automated assessment of epicardial fat volume on CCT (21), total pericardial fat was independently associated with AF recurrence in patients with persistent AF but not in paroxysmal AF. Reasons for differences in our findings may include the fact that prior investigators did not measure atrial iLAEAT, instead focusing on overall epicardial fat burden. This is significant because prior studies have shown that periatrial fat is more tightly linked to the incidence and recurrence of AF than periventricular fat (22, 23).

In our study, we indexed LA adipose tissue to BSA to reduce the known relations of gender and total body size on LA structure and visceral adiposity (34). This is akin to the approach proposed for LA volumetric assessment in the American Society of Echocardiography guidelines for chamber quantification (27). Our stratified analysis shows that iLAEAT predicts AF recurrence among individuals with a BMI greater than 30 kg/m^2^. This may relate to the association between visceral adiposity, metabolic dysregulation, and AF recurrence (35, 36). Our findings show that iLAEAT is an independent predictor of AF burden and recurrence that may be clinically useful. Our hypothesis is supported by other investigations showing that indexed epicardial fat volume is tightly associated with major adverse cardiovascular events as well as coronary plaque volume (37, 38).

### Strengths and limitations

Our study demonstrates that iLAEAT is a novel parameter that can be easily obtained from routinely obtained pre-ablation CT scans. This was performed in under 2 min by an experienced operator as opposed to a processing time of 5 to 11 min reported for volumetric measurements (10). Its serves as a readily available discriminatory tool for AF recurrence which requires minimal operator training and no additional cost. Thus, iLAEAT may be a powerful additional tool in guiding therapeutic decision making regarding CA in AF patients. This study leverages robust data from a comprehensive AF registry containing accurate, up to date and systematically collected clinical, echocardiographic and rigorously adjudicated outcomes. In addition, we systematically measured echocardiographic and CCT phenotypes using a standardized technique blinded to AF status or outcomes. However, this study has a few limitations. This is a retrospective analysis of prospectively gathered data which limits the assessment of causality. We were unable to perform age, gender and BMI matched case-control analyses. Also, we did not measure anthropologic metrics and biochemical markers such as interleukins, tumor necrosis factor-alpha, and hemoglobin A1C which may have added to our understanding of the mechanisms underlying the proposed association. We recognize that this is a moderately sized cohort and may be under-powered to detect certain differences. Validation of these findings in a larger cohort is warranted. Volumetric assessment of epicardial fat was not available. However, the cost, need for specialized software and significant time associated with obtaining the measurements using cumbersome software makes it impractical to obtain fat volumes in every patient. On the contrary, many centers routinely obtain Cardiac CT scans for defining pulmonary vein anatomy. Hence, we believe that routine use of iLAEAT to guide prognosis is more feasible.

## Conclusions

In this study, we demonstrated that iLEAT is associated with adverse cardiac structure, function, as well as AF type and AF recurrence after catheter ablation. Since CCT is routinely obtained in patients prior to AF ablation, measurement of iLAEAT may help risk stratification for AF prognosis beyond traditional clinical risk factors. Furthermore, our findings suggest a strong pathophysiological connection between epicardial fat and atrial remodeling. Research is needed to examine the underlying biochemical, electrical and structural mechanisms underlie the strong associations observed in our study between visceral, epicardial adipose tissue and AF.

## Author contributions

SS conceptualized, designed and implemented the study. SS made CT measurements of epicardial adipose tissue, and echocardiographic measurements, drafted the initial manuscript and approved the final manuscript as submitted. MS contributed to the design of the study, drafting of the initial manuscript and approved the final manuscript as submitted. BH made CT measurements of epicardial adipose tissue and contributed to the drafting of the initial manuscript and approved the final manuscript as submitted. DL contributed to the design of the study and performed statistical analysis and also contributed to drafting of the initial manuscript and approved the final manuscript as submitted. SD contributed to the design and implementation of the study and also contributed to drafting of the initial manuscript and approved the final manuscript as submitted. GA contributed to the design and implementation of the study. GA also contributed to drafting of the initial manuscript and approved the final manuscript as submitted. TF conceptualized, designed and supported the implementation of the study. TF contributed to the drafting of the initial manuscript and approved the final manuscript as submitted. DM conceptualized, designed and supported the implementation of the study. DM contributed to the drafting of the initial manuscript and approved the final manuscript as submitted.

### Conflict of interest statement

The authors declare that the research was conducted in the absence of any commercial or financial relationships that could be construed as a potential conflict of interest.

## References

[1] Iacobellis G Ribaudo MC Assael F Vecci E Tiberti C Zappaterrano A. Echocardiographic epicardial adipose tissue is related to anthropometric and clinical parameters of metabolic syndrome: a new indicator of cardiovascular risk. J Clin Endocrinol Metab. (2003) 88:5163–8. 10.1210/jc.2003-030698 14602744

[2] Tamarappoo B Dey D Shmilovich H Nakazato R Gransar H Cheng VY. Increased pericardial fat volume measured from noncontrast CT predicts myocardial ischemia by SPECT. JACC Cardiovasc. Imaging (2010) 3:1104–12. 10.1016/j.jcmg.2010.07.014 21070997 PMC3057558

[3] Dey D Wong ND Tamarappoo B Nakazato R Gransar H Cheng VY. Computer-aided non-contrast CT based quantification of pericardial and thoracic fat and their associations with coronary calcium and metabolic syndrome. Atherosclerosis (2010) 209:136–41. 10.1016/j.atherosclerosis.2009.08.032 19748623 PMC2830349

[4] Iacobellis G Sharma AM Pellicelli AM Grisorio B Barbarini G Barbaro G. Epicardial adipose tissue is related to carotid intima-media thickness and visceral adiposity in HIV-infected patients with highly active antiretroviral therapy-associated metabolic syndrome. Curr HIV Res. (2007) 5:275–9. 10.2174/157016207780077084 17346142

[5] Doesch C Haghi D Fluchter S Suselback T Schoenberg SO Michaely H. Epicardial adipose tissue in patients with heart failure. J Cardiovasc Magn Reson. (2010) 12:40. 10.1186/1532-429X-12-40 20624277 PMC2914772

[6] Thanassoulis G Massaro JM O'Donnell CJ Hoffmann U Levy D Ellinor PT. Pericardial fat is associated with prevalent atrial fibrillation: the Framingham Heart Study. Circ Arrhythm Electrophysiol. (2010) 3:345–50. 10.1161/CIRCEP.109.912055 20558845 PMC2953855

[7] Fox CS Massaro JM Hoffmann U Pou KM Maurovich-Horvat P Liu CY. Abdominal visceral and subcutaneous adipose tissue compartments: association with metabolic risk factors in the Framingham Heart Study. Circulation (2007) 116:39–48. 10.1161/CIRCULATIONAHA.106.675355 17576866

[8] Fitzgibbons TP Czech M P. Epicardial and perivascular adipose tissues and their influence on cardiovascular disease: basic mechanisms and clinical associations. J Am Heart Assoc. (2014) 3:e000582 10.1161/JAHA.113.000582 24595191 PMC4187500

[9] Iacobellis G Assael F Ribaudo MC Zappaterreno A Alessi G Di Mario U. Epicardial fat from echocardiography: a new method for visceral adipose tissue prediction. Obes Res. (2003) 11:304–10. 10.1038/oby.2003.45 12582228

[10] Marwan M Achenbach S. Quantification of epicardial fat by computed tomography: why, when and how? J Cardiovasc Comput Tomogr. (2013) 7:3–10. 10.1016/j.jcct.2013.01.002 23452994

[11] Wong CX Abed HS Molaee P Nelson AJ Brooks AG Sharma G. Pericardial fat is associated with atrial fibrillation severity and ablation outcome. J Am Coll Cardiol. (2011) 57:1745–51. 10.1016/j.jacc.2010.11.045 21511110

[12] Pou KM Massaro JM Hoffmann U Lieb K Vasan RS O'Donnell CJ. Patterns of abdominal fat distribution: the Framingham Heart Study. Diabetes Care (2009) 32:481–5. 10.2337/dc08-1359 19074995 PMC2646033

[13] Sacks HS Fain JN. Human epicardial adipose tissue: a review. Am Heart J. (2007) 153:907–17. 10.1016/j.ahj.2007.03.019 17540190

[14] McManus DD Lyass A Ingelsson E Massaro JM Meigs JB Aragam J. Relations of circulating resistin and adiponectin and cardiac structure and function: the Framingham Offspring study. Obesity (2012) 20:1882–6. 10.1038/oby.2011.32 21350435 PMC3716016

[15] Marcus GM Smith LM Ordovas K Scheinman MM Kim AM Badhwar N. Intracardiac and extracardiac markers of inflammation during atrial fibrillation. Heart Rhythm (2010) 7:149–54. 10.1016/j.hrthm.2009.10.004 20022819 PMC2900773

[16] Shin SY Yong HS Lim HE Na JO Choi CU Kim SH. Total and interatrial epicardial adipose tissues are independently associated with left atrial remodeling in patients with atrial fibrillation. J Cardiovasc Electrophysiol. (2011) 22:647–55. 10.1111/j.1540-8167.2010.01993.x 21235672

[17] Nguyen BL Fishbein MC Chen LS Chen PS Masroor S. Histopathological substrate for chronic atrial fibrillation in humans. Heart Rhythm (2009) 6:454–60. 10.1016/j.hrthm.2009.01.010 19324302 PMC2662134

[18] Venteclef N Guglielmi V Balse E Gaborit B Cotillard A Atassi F. Human epicardial adipose tissue induces fibrosis of the atrial myocardium through the secretion of adipo-fibrokines. Eur Heart J. (2015) 36:795–805. 10.1093/eurheartj/eht099 23525094

[19] Schauerte P Scherlag BJ Pitha J Scherlag MA Reynolds D Lazzara R. Catheter ablation of cardiac autonomic nerves for prevention of vagal atrial fibrillation. Circulation (2000) 102:2774–80. 10.1161/01.CIR.102.22.2774 11094046

[20] Chiou CW Eble JN Zipes DP. Efferent vagal innervation of canine atria and sinus and atrioventricular nodes: the third fat pad. Circulation (1997) 95:2573–84. 10.1161/01.CIR.95.11.2573 9184589

[21] Kim TH Park J Park JK Uhm JS Joung B Lee MH Pericardial fat volume is associated with clinical recurrence after catheter ablation for persistent atrial fibrillation but not paroxysmal atrial fibrillation: an analysis of over 600 patients. Int J Cardiol. (2014) 176:841–6. 10.1016/j.ijcard.2014.08.008 25176630

[22] Batal O Schoenhagen P Shao M Ayyad AE Van Wagoner DR Halliburton SS. Left atrial epicardial adiposity and atrial fibrillation. Circ Arrhythm Electrophysiol. (2010) 3:230–6. 10.1161/CIRCEP.110.957241 20504944 PMC2974566

[23] Kocyigit D Gurses KM Yalcin MU Turk G Evranos B Yorgun H. Periatrial epicardial adipose tissue thickness is an independent predictor of atrial fibrillation recurrence after cryoballoon-based pulmonary vein isolation. J Cardiovasc Comput Tomogr. (2015) 9:295–302. 10.1016/j.jcct.2015.03.011 26003920

[24] Stojanovska J Kazerooni EA Sinno M Gross BH Wacharotone K Patel S. Increased epicardial fat is independently associated with the presence and chronicity of atrial fibrillation and radiofrequency ablation outcome. Eur Radiol. (2015) 25:2298–309. 10.1007/s00330-015-3643-1 25764090

[25] Lip G Nieuwlaat R Pisters R Lane DA Crijns HJ. Refining clinical risk stratification for predicting stroke and thromboembolism in atrial fibrillation using a novel risk factor-based approach. Chest (2010) 137:263–72. 10.1378/chest.09-1584 19762550

[26] Mosteller RD. Simplified calculation of body-surface area. N Eng J Med. (1987) 317:1098. 10.1056/NEJM198710223171717 3657876

[27] Lang RM Bierig M Devereux RB Flachskampf FA Foster E Pellikka PA. American Society of Echocardiography's guidelines and standards committee European Association of Echocardiography recommendations for chamber quantification: a report from the American Society of Echocardiography's Guidelines and Standards Committee and the chamber quantification writing group, developed in conjunction with the European Association of Echocardiography, a branch of the European Society of Cardiology. J Am Soc Echocardiogr. (2005) 18:1440–63. 10.1016/j.echo.2005.10.005 16376782

[28] Nagueh SF Appleton CP Gillebert TC Marino PN Oh JK Smiseth OA. Recommendations for the evaluation of left ventricular diastolic function by echocardiography. J Am Soc Echocardiogr.(2009) 22:107–33. 10.1016/j.echo.2008.11.023 19187853

[29] Rudski LG Lai WW Afilalo J Hua L Handschumacher MD Chandrasekaran K. Guidelines for the echocardiographic assessment of the right heart in adults: a report from the American Society of Echocardiography endorsed by the European Association of Echocardiography, a registered branch of the European Society of Cardiology, and the Canadian Society of Echocardiography. J Am Soc Echocardiogr. (2010) 23:685–713. 10.1016/j.echo.2010.05.010 20620859

[30] Calkins H Kuck KH Cappato R Brugada J Camm AJ Chen SA HRS/EHRA/ECAS expert consensus statement on catheter and surgical ablation of atrial fibrillation. Heart Rhythm (2012) 9:632–96.e21. 10.1016/j.hrthm.2011.12.016 22386883

[31] Friedman DJ Wang M Meiggs JB Hoffman U Massaro JM Fox CS. Pericardial fat is associated with atrial conduction: the Framingham Heart Study. J Am. Heart Assoc. (2014) 3:e000477. 10.1161/JAHA.113.000477 24595189 PMC4187474

[32] Kanazawa H Yamabe H Enomoto K Koyama J Morihisa K Hoshiyama T. Importance of pericardial fat in the formation of complex fractionated atrial electrogram region in atrial fibrillation. Int J Cardiol. (2014) 174:557–64. 10.1016/j.ijcard.2014.04.135 24834998

[33] Nagashima K Okumura Y Watanabe I Nakai T Ohkubo K Kofune M Does location of epicardial adipose tissue correspond to endocardial high dominant frequency or complex fractionated atrial electrogram sites during atrial fibrillation? Circ Arrhythm Electrophysiol. (2012) 5:676–83. 10.1161/CIRCEP.112.971200 22772897

[34] Pritchett AM Jacobsen SJ Mahoney DW Rodeheffer RJ Bailey KR Redfield MM. Left atrial volume as an index of left atrial size: a population-based study. J Am Coll Cardiol. (2003) 41:1036–43. 10.1016/S0735-109702981-9 12651054

[35] Mohanty S Mohanty P Di Biase L Bai R Pump A Santangeli P. Impact of metabolic syndrome on procedural outcomes in patients with atrial fibrillation undergoing catheter ablation. J Am Coll Cardiol. (2012) 59:1295–301. 10.1016/j.jacc.2011.11.051 22464257

[36] Berkowitsch A Kuniss M Greiss H Wójcik M Zaltsberg S Lehinant S. Impact of impaired renal function and metabolic syndrome on the recurrence of atrial fibrillation after catheter ablation: a long term follow-up. Pacing Clin Electrophysiol. (2012) 35:532–43 10.1111/j.1540-8159.2012.03350.x 22428529

[37] Shmilovich H Dey D Cheng VY Rajani R Nakazato R Otaki Y. Threshold for the upper normal limit of indexed epicardial fat volume: derivation in a healthy population and validation in an outcome-based study. Am J Cardiol. (2011). 108:1680–5. 10.1016/j.amjcard.2011.07.031 21880291 PMC3215795

[38] You S Sun JS Park SY Baek Y Kang DK. Relationship between indexed epicardial fat volume and coronary plaque volume assessed by cardiac multidetector CT. Medicine (2016) 95:e4164. 10.1097/MD.0000000000004164 27399137 PMC5058866

